# Machine-Learning Monitoring System for Predicting Mortality Among Patients With Noncancer End-Stage Liver Disease: Retrospective Study

**DOI:** 10.2196/24305

**Published:** 2020-10-30

**Authors:** Yu-Jiun Lin, Ray-Jade Chen, Jui-Hsiang Tang, Cheng-Sheng Yu, Jenny L Wu, Li-Chuan Chen, Shy-Shin Chang

**Affiliations:** 1 Department of Family Medicine School of Medicine, College of Medicine Taipei Medical University Taipei Taiwan; 2 Department of Family Medicine Taipei Medical University Hospital Taipei Taiwan; 3 Department of Surgery, School of Medicine College of Medicine Taipei Medical University Taipei Taiwan; 4 Division of General Surgery Department of Surgery Taipei Medical University Hospital Taipei Taiwan; 5 Division of Gastroenterology and Hepatology Department of Internal Medicine Taipei Medical University Hospital Taipei Taiwan; 6 Department of Community and Preventive Medicine Taipei Medical University Hospital Taipei Taiwan; 7 School of Gerontology Health Management College of Nursing Taipei Medical University Taipei Taiwan

**Keywords:** visualized clustering heatmap, machine learning, ensemble learning, noncancer-related end-stage liver disease, data analysis, medical information system

## Abstract

**Background:**

Patients with end-stage liver disease (ESLD) have limited treatment options and have a deteriorated quality of life with an uncertain prognosis. Early identification of ESLD patients with a poor prognosis is valuable, especially for palliative care. However, it is difficult to predict ESLD patients that require either acute care or palliative care.

**Objective:**

We sought to create a machine-learning monitoring system that can predict mortality or classify ESLD patients. Several machine-learning models with visualized graphs, decision trees, ensemble learning, and clustering were assessed.

**Methods:**

A retrospective cohort study was conducted using electronic medical records of patients from Wan Fang Hospital and Taipei Medical University Hospital. A total of 1214 patients from Wan Fang Hospital were used to establish a dataset for training and 689 patients from Taipei Medical University Hospital were used as a validation set.

**Results:**

The overall mortality rate of patients in the training set and validation set was 28.3% (257/907) and 22.6% (145/643), respectively. In traditional clinical scoring models, prothrombin time-international normalized ratio, which was significant in the Cox regression (*P*<.001, hazard ratio 1.288), had a prominent influence on predicting mortality, and the area under the receiver operating characteristic (ROC) curve reached approximately 0.75. In supervised machine-learning models, the concordance statistic of ROC curves reached 0.852 for the random forest model and reached 0.833 for the adaptive boosting model. Blood urea nitrogen, bilirubin, and sodium were regarded as critical factors for predicting mortality. Creatinine, hemoglobin, and albumin were also significant mortality predictors. In unsupervised learning models, hierarchical clustering analysis could accurately group acute death patients and palliative care patients into different clusters from patients in the survival group.

**Conclusions:**

Medical artificial intelligence has become a cutting-edge tool in clinical medicine, as it has been found to have predictive ability in several diseases. The machine-learning monitoring system developed in this study involves multifaceted analyses, which include various aspects for evaluation and diagnosis. This strength makes the clinical results more objective and reliable. Moreover, the visualized interface in this system offers more intelligible outcomes. Therefore, this machine-learning monitoring system provides a comprehensive approach for assessing patient condition, and may help to classify acute death patients and palliative care patients. Upon further validation and improvement, the system may be used to help physicians in the management of ESLD patients.

## Introduction

End-stage liver disease (ESLD) is a major public health problem. It is estimated that 1 million patients died from ESLD globally in 2010, accounting for approximately 2% of all deaths [1-6]. Despite improvements in health care, mortality due to ESLD increased by 65% from 1999 to 2016 [7]. Patients with ESLD have limited treatment options and have a deteriorated quality of life with an uncertain prognosis [8]. Early identification of patients with ESLD who have a poor prognosis is fundamental for palliative care.

Several ESLD risk prediction models have been developed using traditional statistical modeling, including the Child-Pugh score [9], model for end-stage liver disease (MELD) [9,10], adjusted MELD scores (eg, MELD-Na score and integrated MELD score) [11-13], albumin-bilirubin score [14], Chronic Liver Failure Consortium (CLIF) Acute Decompensation Score [15], CLIF Sequential Organ Failure Score [16], CLIF Consortium Acute-on-Chronic Liver Failure Score [17], and a novel score recently developed by our group [18]. Unfortunately, these prediction scores were all found to have poor discrimination between survival and death [19-22]. In addition, these traditional risk scores cannot differentiate patients that need acute care or palliative care.

Machine learning, which is the use of computer algorithms that improve automatically through experience, has recently been utilized in disease diagnosis and prediction. In fact, several studies found that machine-learning models have either better or similar performances as traditional statistical modeling approaches [23-26]. Supervised machine-learning models can predict binary disease outcomes, but the prediction accuracy drops when the disease outcome involves several stages. Unsupervised machine-learning models have been successfully utilized to classify diseases that have several stages, such as chronic kidney diseases [27,28]. ESLD is a progressive disease that requires either acute or palliative care. Therefore, the goal of this study was to utilize both supervised and unsupervised machine learning to improve the care of ESLD patients. Specifically, we aimed to create a machine-learning monitoring system that combines several machine-learning models with visualized graphs, including decision trees, ensemble learning methods, and clustering, to predict the mortality of ESLD patients.

## Methods

### Study Participants and Data Collection

**Figure 1:**
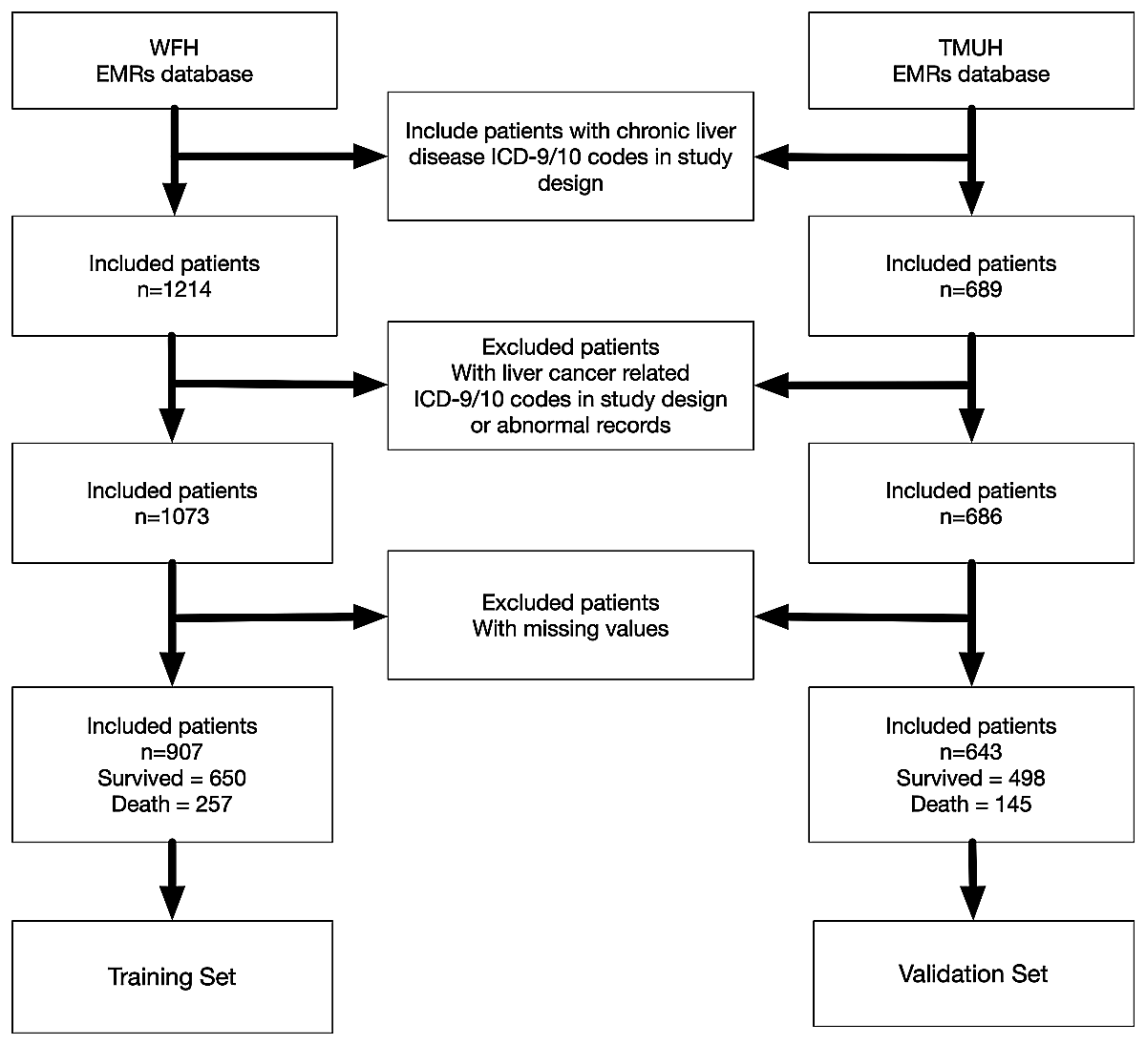
Study flowchart depicting the series of procedures from enrollment to outcome for data collection from patients with noncancer-related end-stage liver disease. WFH: Wan Fang Hospital; TMUH: Taipei Medical University Hospital; EMR: electronic medical record; ICD: International Classification of Diseases.

We conducted a retrospective cohort study using the electronic medical records (EMRs) of patients from Wan Fang Hospital and Taipei Medical University (TMU) Hospital (Figure 1). The training dataset comprised patients from Wan Fang Hospital only, whereas the validation set comprised patients from TMU Hospital. By validating our results in different settings, we tried to ensure that the models developed remained valid and robust in different hospitals.

The study included all adults (aged>18 years) who were diagnosed as having chronic liver diseases with or without related complications of spontaneous bacterial peritonitis, hepatic coma, and esophageal varices (Table 1). In addition, included patients needed to have laboratory EMR data available within 24 hours of admission. Exclusion criteria included pregnancy, cancer, or had a liver transplantation.

Wan Fang Hospital and TMU Hospital are both managed by TMU. The clinical database of TMU includes the EMRs of the two hospitals. The study was approved by the TMU Institutional Review Board (approval number: N202002023) and was conducted in accordance with the Helsinki Declaration.

**Table 1:** International Classification of Diseases (ICD)-9-Clinical Modification (CM) and ICD-10-CM codes for noncancer end-stage liver disease (ESLD).

Diseases	Included in noncancer ESLD	ICD-9-CM code	ICD-10-CM
Cirrhosis	Yes	571.xx	K74.xx
Hepatic coma	Yes	070.xx; 572.xx	K70.xx
Spontaneous bacterial peritonitis	Yes	567.xx	K65.2
Esophageal varices	Yes	456.xx	I85.xx
Malignant neoplasm of the liver	No	155.xx	C22.xx; Z51.12
Liver transplant	No	996.82	Z94.4;T86.4x

### Study Overview and Design

**Figure 2:**
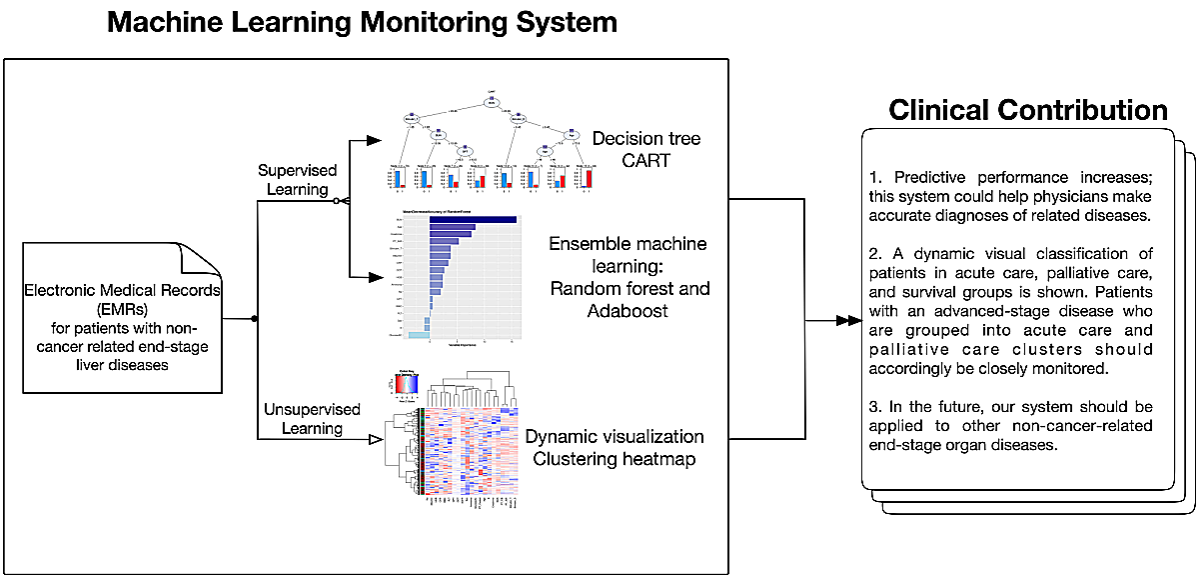
Flowchart depicting the structure of the machine learning medical system. CART: classification and regression tree; Adaboost: adaptive boosting.

The aim of this study was to develop noncancerous liver disease survival prediction models using both traditional statistical modeling approaches and machine-learning approaches (Figure 2). Both supervised and unsupervised machine-learning models were investigated in parallel. For supervised machine learning, the main output was to identify the model with the best survival prediction performance via comparison of the concordance statistic (c-statistic). For unsupervised learning, the main output was the dynamic visualization of ESLD patients to aid in the palliative care of patients. Therefore, ESLD patients were classified into acute death, palliative care, and survived. Acute death was defined as death within 30 days and palliative care was defined as death within 1-9 months from the date of first admission. Mortality was defined using EMR codes related to patient death or critical illness and discharge against medical advice.

Data input was based on the literature and the physician’s clinical judgment. For example, the following biochemical parameters associated with chronic liver disease were recorded: ammonia, albumin, blood urea nitrogen (BUN), complete blood count, C-reactive protein (CRP), creatinine, glutamic-pyruvic transaminase, prothrombin time (PT) and international normalized ratio (INR), glutamic-oxaloacetic transaminase, serum sodium, serum potassium, and total bilirubin.

### Statistical Analysis

Continuous variables were compared by the nonparametric Wilcoxon rank-sum test and categorical variables were compared by the chi-square test.

An initial bivariate analysis was performed to identify significant associations between mortality and all variables available in the study. Significant variables (*P*<.10) were subsequently tested in a stepwise multivariate logistic regression and stepwise Cox proportional hazards regression to identify independent predictors of mortality (*P*<.05). The final model for the stepwise regressions was selected as that with the lowest Akaike information criterion.

The validation dataset was used to compare the performances among all models. Performance was assessed according to comparison of receiver operating characteristic (ROC) curves for the different machine-learning models, including random forest with the MELD score, MELD-Na score, and our novel score [18].

All statistical analyses were performed using R (version 3.6.1) and SAS Enterprise Guide (version 7.1) software. For all analyses, *P*<.05 represented statistical significance.

### Machine-Learning Techniques

Machine learning is a statistical-based model that computer systems use to perform a task without using explicit instructions or inferences [29]. In general, machine-learning algorithms can be subdivided into either supervised or unsupervised learning algorithms. Supervised learning involves building a mathematical model of a dataset, termed training data, that contains the inputs and desired outputs known as a supervisory signal. The model is then tested using a validation set. Supervised learning algorithms involve classification and regression. The supervised machine-learning tools utilized in this study included linear discriminant analysis (LDA), support vector machine (SVM), naive Bayes classifier, decision tree, random forest, and adaptive boosting. By contrast, for unsupervised learning, a dataset is taken that contains only inputs and the structure is identified in the data, such as through grouping and clustering.

#### LDA

LDA is commonly used in multivariate statistical analysis, as it can find a linear combination of features that separates two groups of objects. Hence, LDA is usually used in classification and dimensionality reduction. In this study, LDA was applied to predict the mortality of patients with chronic liver diseases using the “MASS” package in R [30].

#### SVM

SVM constructs a hyperplane in a high-dimensional space for classification and regression. The ideal hyperplane will have the largest distance of margins that separates the two groups of objects. SVM is a nonprobabilistic binary classifier, as it can divide two groups of subjects and can assign new events to one group or the other [31].

#### Naive Bayes Classifiers

Naïve Bayes classifier is based on the Bayes’ theorem, with an independence assumption among these features as probabilistic classifiers. Naïve Bayes can be considered a conditional probability model, which assigns a class label according to the maximum a posteriori decision rule [32].

#### Decision Tree

A decision tree model is a nonparametric and effective machine-learning model. Classification and regression tree (CART) is a typical tree-based model that can predict either a continuous (regression tree) or categorical (classification tree) outcome, and visualizes the decision rule [33]. In decision tree, the Gini index (Equation 1) is used to decide the nodes on a decision branch where *p_i_* represents the relative frequency of the class that is being observed in the dataset and *c* represents the number of classes. The process of the CART algorithm at each node for classification is as follows: (1) construct a split condition, (2) select a split condition, (3) calculate the impurity by the Gini index (Equation 1), (4) execute steps 2 to 4 until the minimum impurity is selection, and (5) construct the classification in the node.

The Gini index is calculated as:


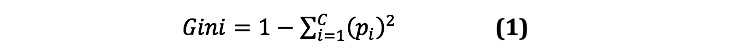


where *p_i_* is the probability of an object being classified to a particular class *i*.

In this study, the tree depth of CART was controlled at 4 (ie, maxdepth=4) in the R package to avoid overfitting based on a previous study [26].

#### Ensemble Learning

Ensemble learning uses multiple learning algorithms to improve machine-learning results, and has generally been found to have better predictive performance than a single model. This is achieved by combining several decision classification and regression tree models [34]. Two types of ensemble learning (random forest and adaptive boosting) were used in this study.

#### Random Decision Tree

Random forest, a random decision tree model, can extract the most relevant variables by performing classification, regression, or other applications based on a decision tree structure. Parallel methods were used to exploit the independence between the base learners because the error can be minimized by averaging. By creating multiple decision trees and combining the output generated by each tree, the model increases predictive power and reduces bias.

The basic single tree model in random forest is a CART using the Gini index as the selection criterion, and the random forest algorithm applies the bagging technique to implement the teamwork of numerous decision tree models, thereby improving the performance of a single model. The bagging procedure is as follows:

(1) Given a training set X = x_1_, x_2_, …, x_n,_ with response Y = y_1_, y_2_, …, y_n_;

(2) For b = 1, 2, …, B, as the repeated bagging time;

(3) Bagging select a random sample X_b_, Y_b_ with replacement of the training set;

(4) Generate a classification tree from X_b_, Y_b_;

(5) Prediction for unseen or testing samples *z* by taking the majority vote from all of the individual classification trees.

The variable importance is determined by the decrease in node impurity, which is weighted by the probability of reaching the node. We determined the node probability by the number of samples that reached the node divided by the total number of samples. Thus, the variable becomes more significant as the value gets higher. The feature importance was implemented by Scikit-learn according to Equations (2) and (3). Assuming a binary tree, Scikit-learn calculates a node’s importance using the Gini index.

*importance (n_i_)= w_i_G_i_ – w_left(i)_G_left(i)_ – w_right(i)_G_right (i)_***(2)**

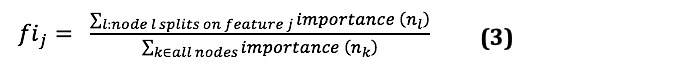


Where *importance (n_i_)* is the importance of node *i*, *w_i_* is the weighted number of samples reaching node *i*, *G_i_* is the impurity value of node *j*, *left(i)* is the left child node from node *i*, *right(i)* is the right child node from node *i*, and *fi_j_* is the importance of feature *j*.

The final feature importance at the random forest is the average over all CART tree models after normalization. That is, the sum of the feature’s importance values on each tree is divided by the total number of trees [35]. We used the R package randomForest in this study [36].

#### Adaptive Boosting

Adaptive boosting is an ensemble learning method in which base learners are generated sequentially. It is also used in conjunction with many weak learners (ie, those with poor-performance classifiers) to improve performance. Improving weak learners and creating an aggregated model to improve model accuracy is crucial for boosting algorithm performance. The output of weak learners is combined into a weighted sum that represents the final output of the boosted classifier. Adaptive boosting is adaptive because the motivation for using sequential methods is exploiting the dependence between the base learners. In addition, the predictive ability can be boosted by weighing previously mislabeled examples with a higher weight. In addition, bagging, a method that combines bootstrapping and aggregating, was used. Because the bootstrap estimate of the data distribution parameters is more accurate and robust, after combining them, a similar method can be used to obtain a classifier with superior properties [37,38]. This study used the “adabag” package for implementing adaptive boosting in R.

#### ROC

We used ROC curves to compare the mortality predictive performances based on the c-statistic, which is equivalent to the area under the curve (AUC) value. The false positive rate (related to specificity) and the true positive rate (also called sensitivity or recall) were calculated for comparison.

#### Heatmap and Clustering

A heatmap was used to visualize the pattern of the clinical variables. The clinical and laboratory data of patients are represented as grids of colors with hierarchical clustering analysis applied for both rows and columns [39]. Patients were separated by Euclidean distance (Equation 4) and clustered using the Ward hierarchical clustering algorithm (Equation 5). Clustering can be upgraded using different similarity measures and clustering algorithms [40]. The heatmap was constructed using the “ggplot” package in R. The Euclidean distance between points *p* and *q* is the length in multidimensional *n*-space calculated as:


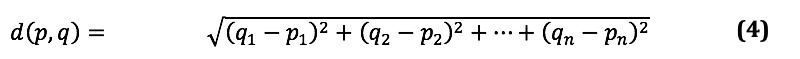


We followed the general agglomerative hierarchical clustering procedure suggested by the Ward method. The criterion for choosing a pair of clusters to merge at each step is based on the Ward minimum variance method, which can be defined and implemented recursively by a Lance–Williams algorithm [41]. The recursive formula gives the updated cluster distances following the pending merge of clusters. We used the following formula to compute the updated cluster distance:

*d*(*C_i_* ∪ *C_j_*, *C_k_*) = *a_i_d*(*C_i_*, *C_k_*) + *a_j_d*(*C_j_*, *C_k_*) + *βd*(*C_i_*, *C_j_*) + *γ*∣*d*(*C_i_*, *C_k_*) – *d*(*C_j_*, *C_k_*)∣ **(5)**

where *d*(*C_i_*, *C_j_*) is the distance defined between cluster *i* and cluster *j*; thus, for each of the metrics we can compute the parameters *α_i_, α_j_*, *β*, and *γ*.

The Ward minimum variance method can be implemented by the Lance–Williams formula as follows:

*d*(*C_i_* ∪ *C_j_*, *C_k_*)= *n_i_*+*n_k_*/*n_i_*+*n_j_*+*n_k_d*(*C_i_*, *C_k_*) + *n_j_*+*n_k_*/*n_i_*+*n_j_*+*n_k_ d*(*C_j_*, *C_k_*) – *n_k_*/*n_i_*+*n_j_*+*n_k_ d*(*C_i_*, *C_j_*) **(6)**,

where *n_i_*, *n_j_*, and *n_k_* is the size of each cluster, *a_i_* is *n_i_*+*n_k_*/*n_i_*+*n_j_*+*n_k_*, *a_j_* is *n_j_*+*n_k_*/*n_i_*+*n_j_*+*n_k_*, *β* is – *n_k_*/*n_i_*+*n_j_*+*n_k_*, and *γ* is 0.

The “ggplot” package provides the function to apply heatmap and hierarchical clustering in R. In the function, “scale” was subject to normalization, and “RowSideColors” were set according to the death outcomes.

## Results

Figure 1 shows an overview of the study participants and Figure 2 gives an overview of the study. Initially, a total of 1214 patients from Wan Fang Hospital were used to establish a dataset for training and 689 patients from TMU Hospital were used for validation. After data preprocessing (ie, excluding cases with abnormal records and liver cancer cases), the overall mortality rate of patients in the training set at Wan Fang Hospital was 28.3% (257/907) and that at TMU Hospital was 22.6% (145/643). Table 2 and Table 3 summarize the baseline characteristics of all patients according to survival status and separated by the training and validation datasets, respectively.

**Table 2:** Demographic and laboratory characteristics of patients with noncancerous liver diseases according to survival status.

Demographic variables^a^		Died (n=257)	Survived (n=650)	*P* value
**Sex, n (%)**				.25
	Male	155 (60.3)	419 (64.5)	
	Female	102 (39.7)	231 (35.5)	
Age (years)		69 (56-82)	60 (50-72)	<.001
Albumin (g/dL)		2.8 (2.5-3.1)	3.1 (2.8-3.6)	<.001
Ammonia (μg/dL)		55 (34-82)	43 (31-68)	.003
Blood urea nitrogen (mg/dL)		29 (18-52)	16 (12-26)	<.001
Total bilirubin (mg/dL)		1.7 (0.9-4.3)	1.3 (0.7-2.5)	<.001
Direct bilirubin (mg/dL)		1 (0.4-2.9)	0.5 (0.2-1.2)	<.001
Creatinine (mg/dL)		1.2 (0.8-2.2)	0.9 (0.7-1.3)	<.001
C-reactive protein (mg/dL)		5.2 (2.8-8.9)	4.1 (1.2-6.3)	<.001
eGFR^b^ (mL/min/1.73 m^2^)		54.6 (33.4-60.5)	62 (57-80)	<.001
Glucose ante cibum (mg/dL)		111 (96-139)	107 (94-139)	.31
Serum GOT^c^ (U/L)		54 (32-94)	40 (26-72)	<.001
Serum GPT^d^ (U/L)		35 (22-59)	33 (21-58)	.42
Hemoglobin (g/dL)		10 (9-11)	12 (10-13)	<.001
Potassium (mEq/L)		4 (3.7-4.4)	3.9 (3.7-4.2)	.001
Sodium (mEq/L)		138 (134-141)	138 (136-139)	.43
Platelets (10^3^/μL)		130 (86-177)	162 (110-217)	<.001
PT^e^ Control (seconds)		10.8 (10.8-12.5)	11.8 (11.8-12.6)	<.001
PT fibrinogen (seconds)		14.8 (12.7-17.1)	13.5 (12.2-15.1)	<.001
PT international normalized ratio		1.3 (1.13-1.54)	1.15 (1.04-1.29)	<.001
Leukocyte count (10^3^/μL)		8.10 (5.99-10.82)	7.02 (5.43-9.28)	<.001

^a^Continuous variables are presented as median (IQR).

^b^eGFR: estimated glomerular filtration rate.

^c^GOT: glutamic-oxaloacetic transaminase.

^d^GPT: glutamic-pyruvic transaminase.

^e^PT: prothrombin time.

**Table 3:** Demographic and laboratory characteristics of patients with noncancerous liver diseases in the training and validation datasets.

Demographic variables^a^		Training (n=907)	Validation (n=643)	*P* value
**Sex, n (%)**				.51
	Male	574 (63.3)	420 (65.3)	
	Female	333 (36.7)	223 (34.7)	
Age (years)		62 (52-75)	61 (51-73)	.11
Albumin (g/dL)		3 (2.7-3.5)	3.3 (3.1-3.7)	<.001
Ammonia (μg/dL)		48 (31-75)	83 (49-116)	<.001
Blood urea nitrogen (mg/dL)		18 (13-33)	16 (12-27)	.003
Total bilirubin (mg/dL)		1.4 (0.8-2.8)	1.5 (0.7-3.1)	.77
Direct bilirubin (mg/dL)		0.6 (0.2-1.7)	1.1 (0.5-2.7)	<.001
Creatinine (mg/dL)		0.9 (0.7-1.5)	0.9 (0.7-1.2)	<.001
C-reactive protein (mg/dL)		4.4 (1.6-7)	3.3 (1.3-4.9)	<.001
eGFR^b^ (mL/min/1.73 m^2^)		60.5 (47.6-73.5)	94.1 (65.1-123.6)	<.001
Glucose ante cibum (mg/dL)		108 (94-139)	121(104-151)	<.001
Serum GOT^c^ (U/L)		43 (27-80)	53 (34-91)	<.001
Serum GPT^d^ (U/L)		34 (21-59)	39 (25-64)	.001
Hemoglobin (g/dL)		11 (10-13)	11 (10-13)	.50
Potassium (mEq/L)		4 (3.7-4.2)	3.9 (3.6-4.2)	.003
Sodium (mEq/L)		138 (135-140)	137 (135-139)	.049
Platelets (10^3^/μL)		154 (102-209)	138 (87-197)	<.001
PT^e^ control (seconds)		11.7 (10.8-12.6)	13.3 (13.2-13.4)	<.001
PT fibrinogen (seconds)		13.8 (12.3-15.6)	15 (13.7-17.4)	<0.001
PT international normalized ratio		1.19 (1.05-1.37)	1.23 (1.08-1.48)	<.001
Leukocyte count (10^3^/μL)		7.38 (5.56-9.71)	6.8 (5.28-8.82)	<.001

^a^Continuous variables are presented as median (IQR).

^b^eGFR: estimated glomerular filtration rate.

^c^GOT: glutamic-oxaloacetic transaminase.

^d^GPT: glutamic-pyruvic transaminase.

^e^PT: prothrombin time.

Table 4 shows the risk factors of mortality-based stepwise multivariate logistic and Cox regression analyses for the training dataset. PT-INR, which was significant in the Cox regression, had a prominent influence on predicting mortality. Moreover, BUN and CRP had significant effects on mortality.

**Table 4:** Significant factors in stepwise multivariate logistic and Cox regression analyses.

Factors		*P* value	Odds ratio/hazard ratio^a^ (95% CI)
**Stepwise multivariate logistic regression**			
	Age	<.001	1.029 (1.017-1.042)
	Albumin	.002	0.590 (0.421-0.827)
	Blood urea nitrogen	.04	1.009 (1.000-1.018)
	C-reactive protein	<.001	1.101 (1.056-1.147)
	Hemoglobin	<.001	0.795 (0.717-0.882)
	Sodium	.02	1.053 (1.007-1.101)
	Platelets	<.001	0.995 (0.992-0.997)
	Total bilirubin	<.001	1.149 (1.087-1.216)
	Leukocyte count	.01	1.075 (1.016-1.137)
**Stepwise Cox regression**			
	Age	.03	1.005 (1.001-1.010)
	Blood urea nitrogen	<.001	1.013 (1.009-1.018)
	Creatinine	.002	0.920 (0.873-0.969)
	C-reactive protein	<.001	1.027 (1.013-1.042)
	PT^b^ international normalized ratio	<.001	1.288 (1.131-1.468)
	Total bilirubin	<.001	1.036 (1.018-1.053)

^a^Odds ratios are reported for logistic regression and hazard ratios are reported for Cox regression.

^b^PT: prothrombin time.

**Figure 3:**
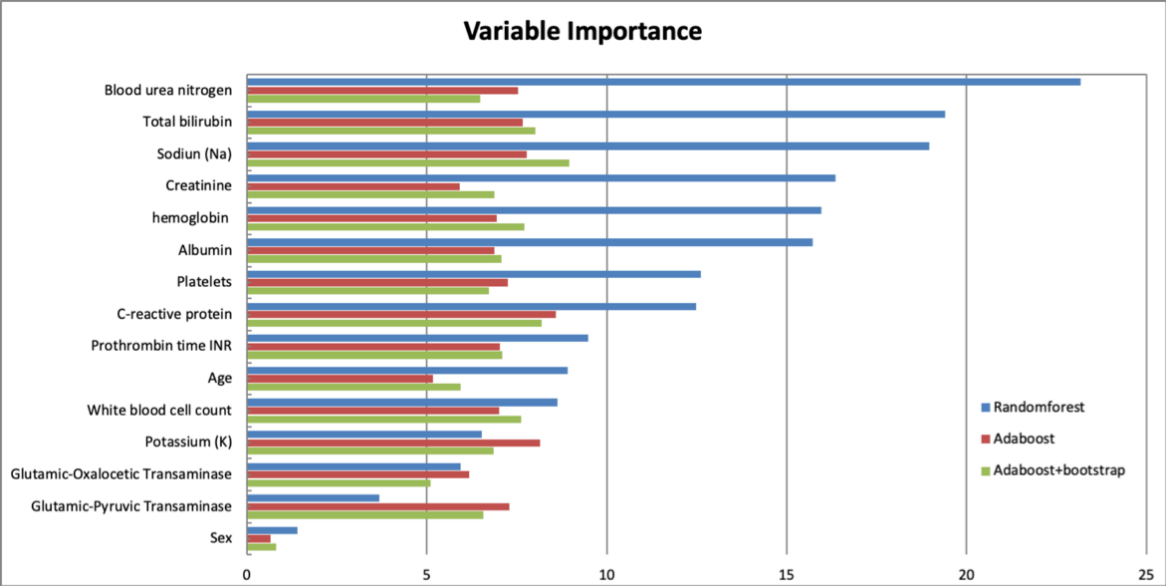
Variable importance ordered by the accuracy of mean decrease in random forest, adaptive boosting (AdaBoost), and AdaBoost + bootstrap. The order of variables is followed by the rank of leading variables in the random forest.

**Figure 4:**
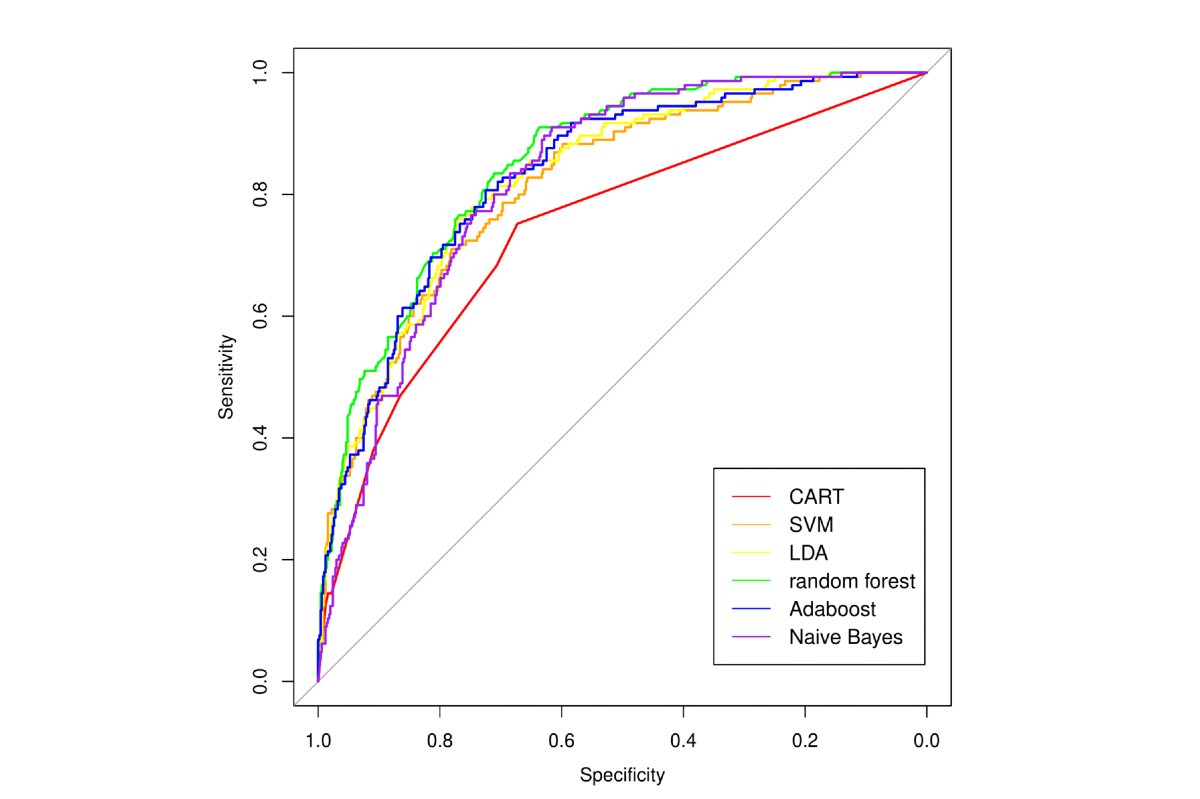
Receiver operating characteristic (ROC) curves with area under the curve (AUC) statistics of classification and regression tree (CART), supervised machine learning (SVM), linear discriminant analysis (LDA), random forest, naive Bayes, and adaptive boosting (Adaboost).

Similar results were obtained using machine-learning methods. Figure 3 shows the variable of importance for random forest and adaptive boosting, which had better performances among all of the supervised machine-learning methods tested (Table 5, Figure 4). BUN was regarded as the primary factor for predicting mortality by both random forest and adaptive boosting models. Creatinine, PT-INR, and bilirubin also emerged as remarkable factors in prediction.

**Table 5:** Performance of different machine-learning models on predicting mortality of patients with noncancer end-stage liver diseases using the validation dataset.

Model	Accuracy	Sensitivity	Specificity	c-statistic^a^
LDA^b^	0.823	0.701	0.839	0.829
SVM^c^	0.818	0.310	0.966	0.817
Naïve Bayes	0.784	0.290	0.928	0.824
CART^d^	0.790	0.379	0.910	0.744
Random Forest	0.824	0.372	0.956	0.852
Adaboost^e^	0.813	0.455	0.918	0.833

^a^c-statistic: concordance statistic of the receiver operating characteristic curve.

^b^LDA: linear discriminant analysis.

^c^SVM: support vector machine.

^d^CART: classification and regression tree.

^e^AdaBoost: adaptive boosting.

**Figure 5:**
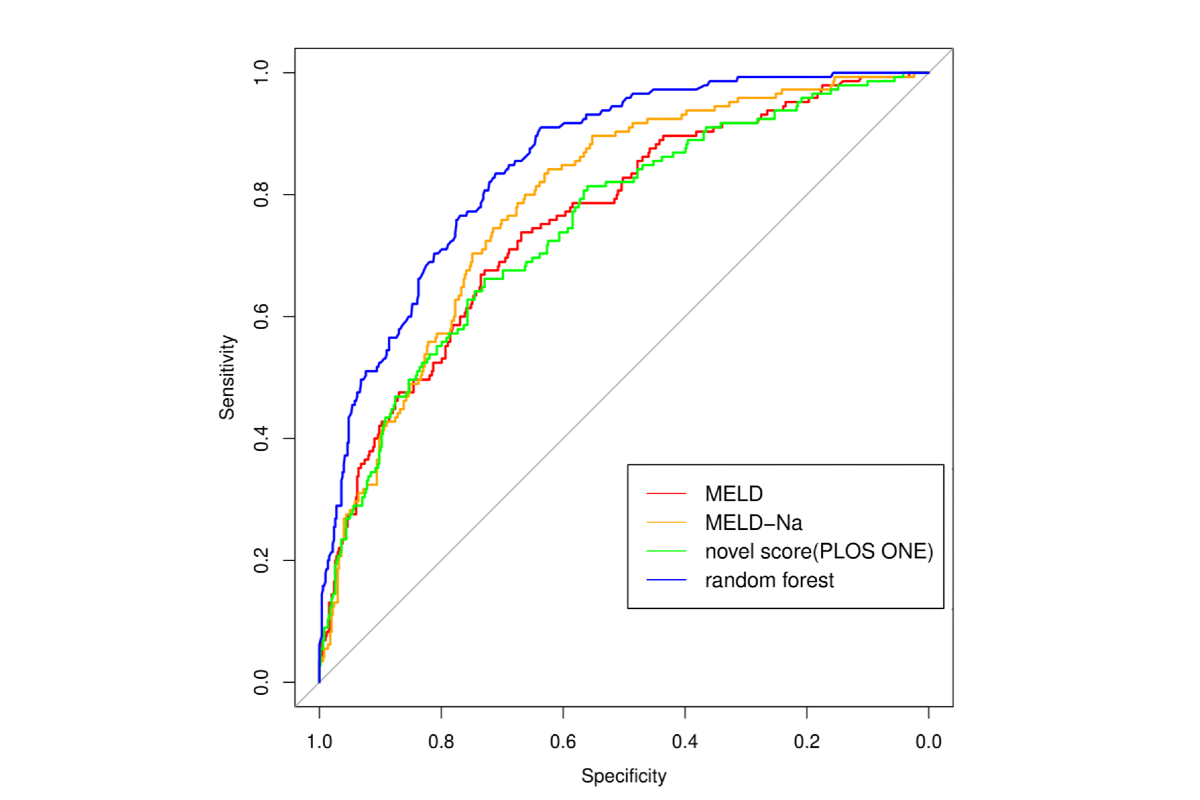
Receiver operator characteristic (ROC) curves with area under the curve (AUC) statistics of random forest, model for end-stage liver disease (MELD) score, MELD-NA score, and novel score.

**Figure 6:**
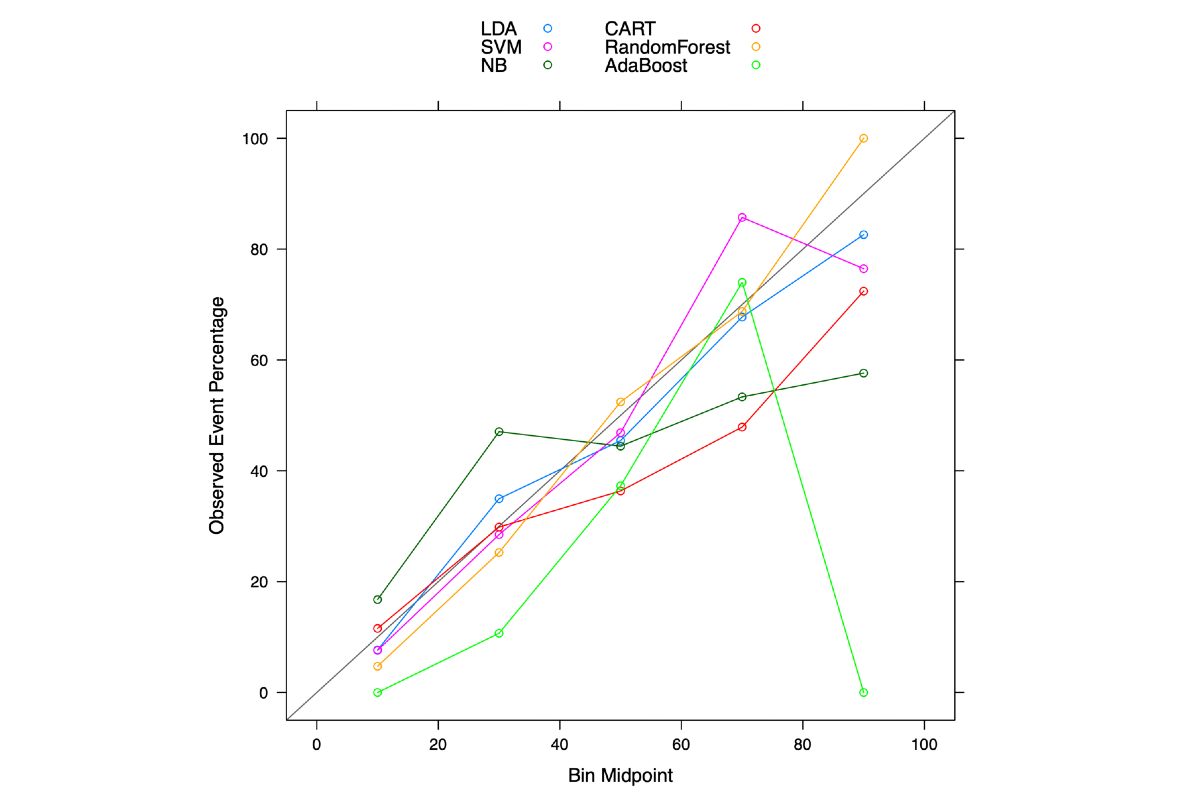
Calibration plots of classification and regression tree (CART), supervised machine learning (SVM), linear discriminant analysis (LDA), random forest, naive Bayes (NB), and adaptive boosting (Adaboost).

Figure 5 compares the ROC curves for mortality prediction between random forest, as the top-performing machine-learning model, with traditional risk scores. It is clear that random forest (blue curve) had better predictability than all traditional risk scores. However, there were overlaps among traditional risk scores, and it is difficult to differentiate the predictive ability of the MELD score (red, AUC=0.76), MELD-Na (orange, AUC=0.79), and novel score (green, AUC=0.75). Figure 6 shows the calibration plots for the different machine-learning models. The calibration plot is divided into 5 risk strata to match the MELD score. In general, most of the points are close to the diagonal, and the random forest model was found to be better calibrated than other machine-learning techniques. Therefore, the majority of machine-learning models showed better performance (according to the c-statistic in Table 5) than the traditional scoring models. The specificity of each machine-learning model was also above 0.80.

**Figure 7:**
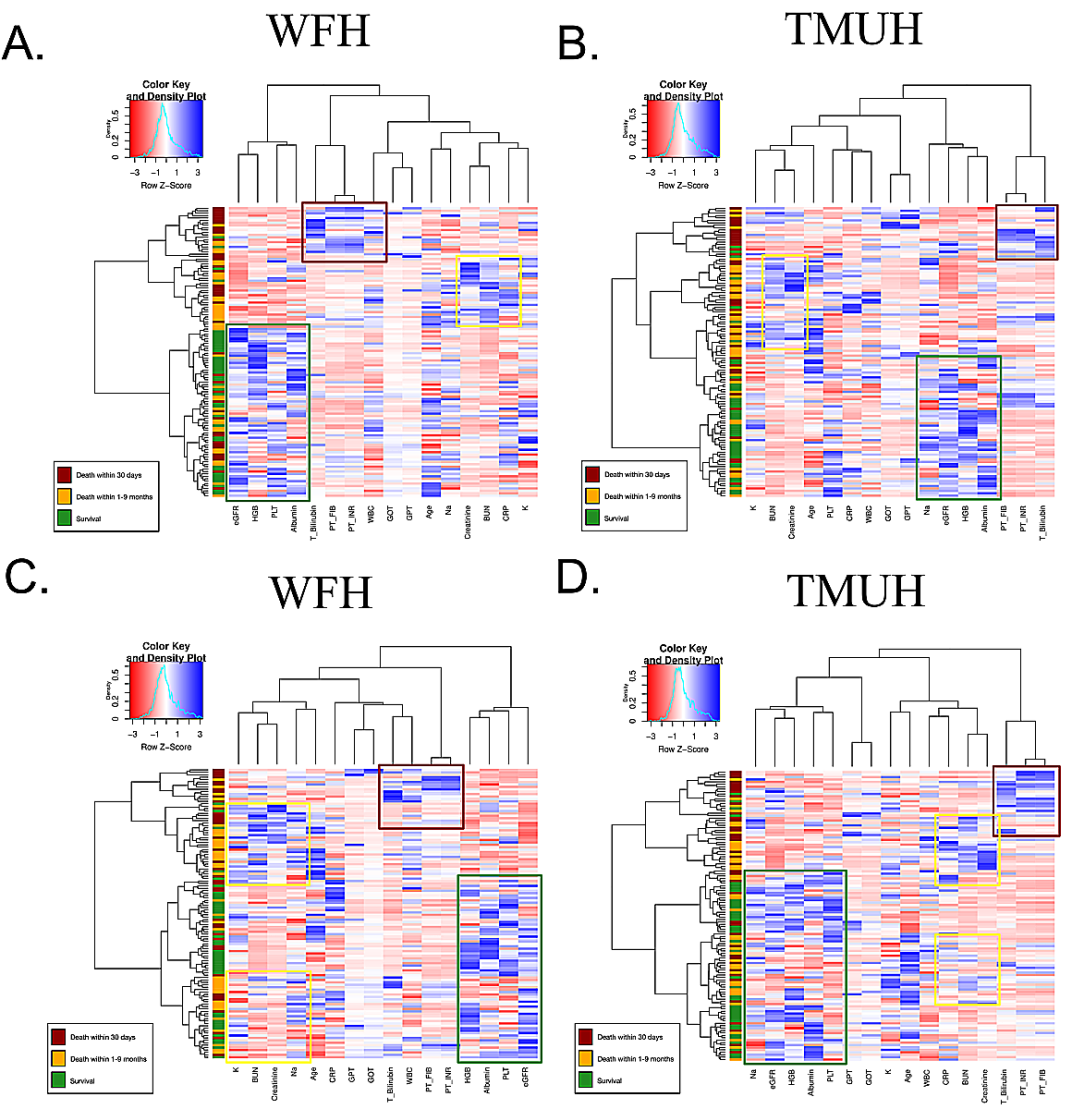
Heatmap showing the classification of acute care (death within 30 days), palliative care (death within 1-9 months), and survival groups in Wan Fang Hospital (WFH) cohort (A and C) and Taipei Medical University Hospital (TMUH) cohort (B and D). BUN: blood urea nitrogen; Bilirubin_T: total bilirubin; CRP: C-reactive protein; eGFR: estimated glomerular filtration rate; GlucoseAC: glucose ante cibum; GOT: serum glutamic-oxaloacetic transaminase; GPT: serum glutamic-pyruvic transaminase; HGB: hemoglobin; K: potassium; Na: sodium; PLT: platelets; PT: prothrombin time; INR: international normalized ratio; WBC: leukocyte count.

In unsupervised machine learning using the heatmap, patients were grouped into death within 30 days (red), death within 1-9 months (yellow), and survival (green) (Figure 7). We found that different clusters had specific color patterns related to laboratory outcomes.

## Discussion

### Principal Findings

A major limitation in traditional statistical modeling is poor predictive ability, especially in nonhomogeneous patients representing several different disease stages. Supervised and unsupervised machine-learning methods are data-driven techniques that have been shown to have either better or similar performances as traditional statistical modeling approaches. In this study, we found that supervised ensemble learning models have better predictive performance than traditional statistical modeling. The AUC of traditional statistical modeling techniques was around 0.75, whereas that of machine-learning techniques was around 0.80. The AUC of the machine-learning technique with the best performance (random forest) was 0.85. In unsupervised learning analysis using hierarchical clustering, ESLD patients were separated into three clusters: acute death, palliative care, and survived.

Traditional regression analysis showed that PT-INR had the highest odds ratio among all of the significant variables in predicting mortality. This is likely because critically ill patients develop hemostatic abnormalities, and PT-INR has been associated with early death among patients with sepsis-associated coagulation disorders [42]. Similar to previous studies, we also found that BUN and CRP can predict mortality in critically ill patients and for those receiving palliative care [43,44]. A prior study also found that total bilirubin is an excellent predictor of short-term (1-week) mortality in patients with chronic liver failure [45]. High bilirubin levels combined with low albumin levels may be used to predict the severity and progression of liver injury [46,47]. Hyperkalemia (high potassium) and hyponatremia (low sodium) have also been found to increase the mortality risk of ESLD patients [48,49].

In the variable of importance analysis using supervised machine-learning models, BUN was regarded as the primary factor for predicting mortality. This result is in line with a recent study showing that a high BUN concentration is robustly associated with adverse outcomes in critically ill patients, and the results remained robust after correction for renal failure [43]. Interestingly, our variable of importance analysis suggested that BUN might be a more crucial parameter for risk stratification than creatinine level in critically ill patients. We hypothesize that BUN could be an independent risk factor for renal failure, which might indicate neurohumoral activation and disturbed protein metabolism.

In the unsupervised learning analysis, ESLD patients were successfully separated into three clusters. We found that leukocyte count, PT, and bilirubin had specific and similar patterns in the acute death cluster when compared with the palliative care and survival clusters. This is likely related to the fact that these parameters are excellent predictors of short-term mortality and were therefore classified with the acute patient group [42,45]. Acute‐on‐chronic liver failure (ACLF) is one of the main causes of mortality of ESLD patients. One of the marked pathophysiological features of ACLF is excessive systemic inflammation, which is mainly manifested by a significant increase in the levels of plasma proinflammatory factors, leukocyte count, and CRP [50,51], as observed in our study.

ESLD patients with hepatorenal syndrome typically have the worst prognosis. There are two types of hepatorenal syndrome: type 1 progresses quickly to renal failure, whereas type 2 evolves slowly. Type 2 hepatorenal syndrome is typically associated with refractory ascites and the 3-month survival is 70% [52]. Although BUN, creatinine, sodium, and potassium are indicators of renal function, considering the progression of hepatorenal syndrome, the clustering heatmap classified these parameters in the palliative care group. Thus, visualization of the monitoring system using machine-learning techniques may furnish health care personnel with sufficient relevant information to manage the treatment of patients with chronic liver diseases.

### Strengths and Limitations

Medical artificial intelligence has become a cutting-edge tool in clinical medicine, as it has been found to have predictive ability in several diseases. The machine-learning monitoring system developed in this study involves multifaceted analyses, which provide various aspects for evaluation and diagnosis. This strength makes the clinical results more objective and reliable. Moreover, the visualized interface in this system offers more intelligible outcomes.

However, this study has several limitations. First, although this study enrolled thousands of ESLD patients, the numbers of ESLD patients who received palliative care or who experienced acute death were small relative to the number of ESLD patients that have survived. Including data from a larger sample of ESLD patients who received palliative care or who died from acute disease will further improve the accuracy of the machine-learning model in differentiating these three types of ESLD patients. Second, this study enrolled only patients in the Taiwanese population, and the external validity of this study with a cohort of different ethnicity remains to be tested. Third, this was a retrospective study, and a cohort study with prospectively enrolled patients is required to determine the usefulness of our system in clinical practice.

### Conclusions and Implications

Our machine-learning monitoring system provides a comprehensive approach for evaluating the condition of patients with ESLD. We found that supervised machine-learning models have better predictive performance than traditional statistical modeling, and the random forest model had the best performance of all models investigated. In addition, our unsupervised machine-learning model may help to differentiate patients that require either acute or palliative care, and may help physicians in their decision in patient treatment. In the future, it will be beneficial to apply our model to several other end-stage organ diseases without the involvement of cancer.

## Abbreviations

ACLF: acute-on-chronic liver failure
AUC: area under the curve
BUN: blood urea nitrogen
CART: classification and regression tree
CLIF: Chronic Liver Failure Consortium
CRP: C-reactive protein
c-statistic: concordance statistic of the receiver operating characteristic curve
EMR: electronic medical record
ESLD: end-stage liver disease
INR: international normalized ratio
LDA: linear discriminant analysis
MELD: model for end-stage liver disease
PT: prothrombin time
ROC: receiver operating characteristic
SVM: support vector machine
TMU: Taipei Medical University
